# Supplementary material for: Isolation of Functional Human MCT Transporters in Saccharomyces cerevisiae
Source: Cells. 2024 Sep 20;13(18):1585. doi: 10.3390/cells13181585 (PMC11430032; doi:10.3390/cells13181585)
Supplement: Supplementary file 1 [file cells-13-01585-s001.zip › cells-3182842-supplementary/cells-3182842-supplementary materials.pdf]

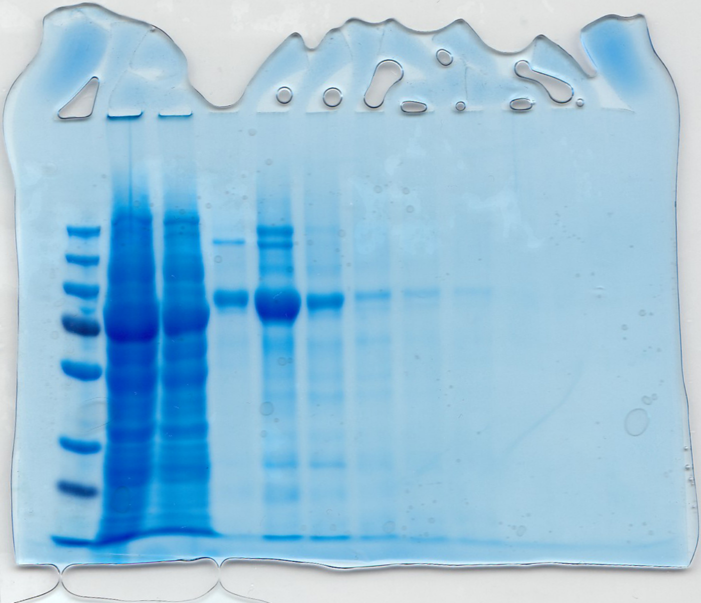

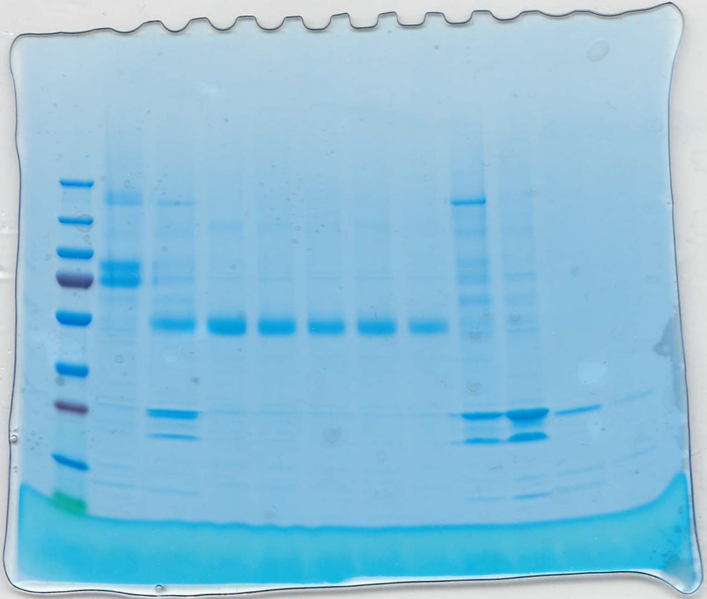

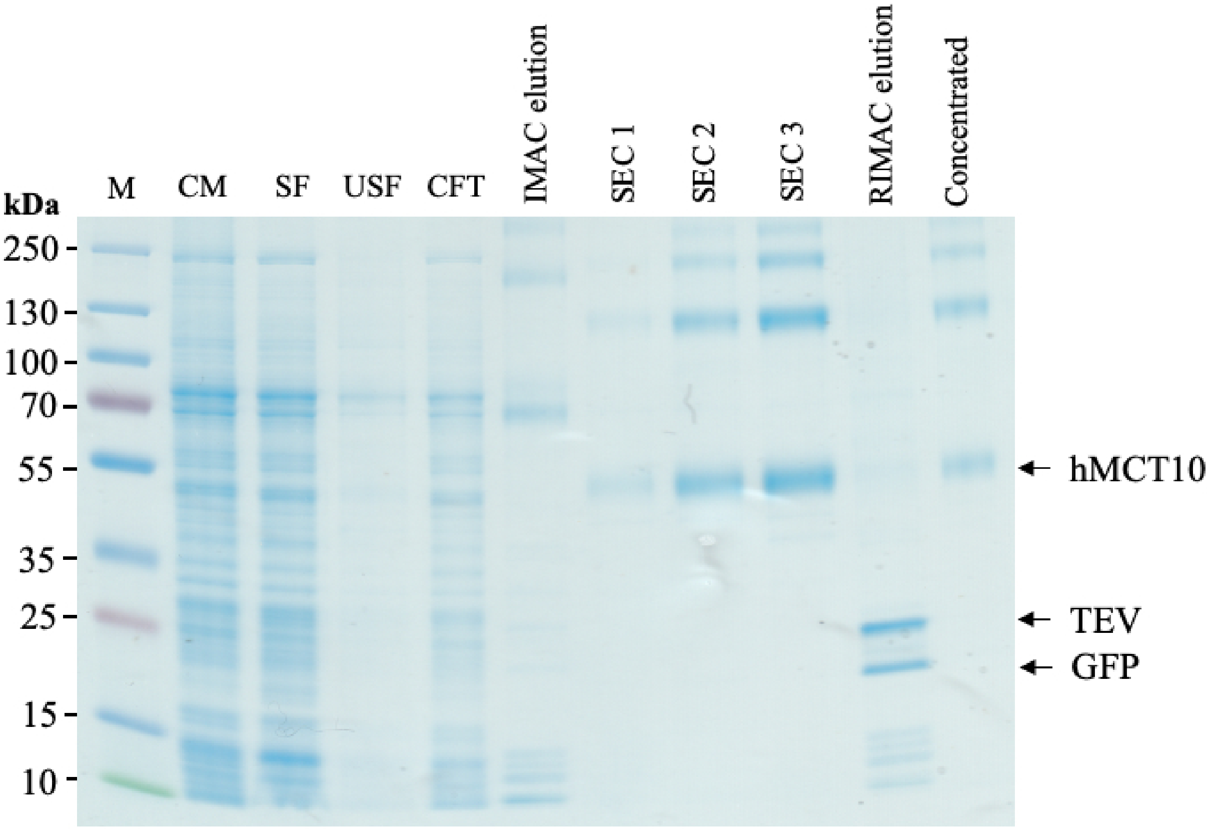

CM = Crude membrane
SF = Souluble fraction
USF = Unsoluble fraction
CTF = Column flowtrough
IMAC = Immobilized metal affinity chromatography
RIMAC = Reverse IMAC
